# Phenotypes of hypertrophic cardiomyopathy. An illustrative review of MRI findings

**DOI:** 10.1007/s13244-018-0656-8

**Published:** 2018-10-22

**Authors:** Rafaela Soler, Cristina Méndez, Esther Rodríguez, Roberto Barriales, Juan Pablo Ochoa, Lorenzo Monserrat

**Affiliations:** 1 Radiology Department, Complexo Hospitalario Universitario A Coruña, Instituto de Investigación Biomédica de A Coruña (INIBIC), Servizo Galego de Saúde (SERGAS), Universidade da Coruña, Xubias de Arriba 86, 15006 A Coruña, Spain; 2 Cardiology Department, Complexo Hospitalario Universitario A Coruña, Instituto de Investigación Biomédica de A Coruña (INIBIC), Servizo Galego de Saúde (SERGAS), Universidade da Coruña, Xubias de Arriba, 84, 15006 A Coruña, Spain

**Keywords:** Hypertrophic cardiomyopathy, Phenotypes, Magnetic resonance, Heart

## Abstract

**Objective:**

The purpose of this article is to review how cardiac MRI provides the clinician with detailed information about the hypertrophic cardiomyopathy (HCM) phenotypes, assessing its morphological and functional consequences.

**Conclusion:**

An understanding of cardiac MRI manifestations of HCM phenotypes will aid early diagnosis recognition and its functional consequences.

**Teaching Points:**

• *The phenotypic variability of HCM expands beyond myocardial hypertrophy, to include morphological and functional manifestations, ranging from subtle anomalies to remodelling of the LV with progressive dilatation and thinning of its wall.*

• *The stages of HCM, which are based on the clinical evidence of disease progression, include subclinical HCM, the classic HCM phenothype, adverse remodelling and overt dysfunction, or end-stage HCM.*

• *Cardiac MRI provides the clinician with detailed information regarding the HCM phenotypes and enables the assessment of its functional consequences.*

## Introduction

Hypertrophic cardiomyopathy (HCM) is a genetic cardiovascular disease, defined by an increase in the left ventricular wall thickness (end-diastolic left ventricular wall thickness ≥ 15 mm or the equivalent relative to the body surface area in children) that is not solely explained by abnormal loading conditions. Lesser degrees of wall thickness (13–14 mm) can also be diagnostic of HCM, particularly when it is identified in family members [1–3]. In up to 60% of adolescents and adults, this disorder is an autosomal dominant trait caused by mutations in cardiac sarcomere protein genes [2, 4, 5].

The microscopic findings of HCM are characteristic with hypertrophy of the myocardial fibres, disorganisation of muscular bundles, interstitial fibrosis and dysfunction of the coronary microvasculature with increased wall thickening that leads to luminal narrowing, silent myocardial ischaemia, and myocardial injury and fibrosis [6].

HCM is characterised by diverse phenotypic expressions and a variable natural progression, which may range from dyspnoea and/or syncope to sudden cardiac death (SCD). HCM is the most common cause of SCD in young athletes [2, 3, 7, 8].

The phenotypic variability of HCM is not limited to only myocardial hypertrophy, but rather includes a set of morphological and functional manifestations, ranging from subtle anomalies to remodelling of the left ventricle (LV) with progressive dilatation and thinning of its wall that evolves to heart failure simulating a restrictive or dilated cardiomyopathy [8–12]. It is important to look for each of these phenotypic expressions to establish the diagnosis and define the importance of the disease [2, 8].

Cardiac MRI has emerged as an imaging modality particularly well-suited to characterise the phenotypic expression of HCM, providing more accurate wall thickness measurements and identifying patients with an increased risk for ventricular arrhythmias and thromboembolic stroke, as well as abnormalities that contribute to LV outflow tract (LVOT) obstruction and differentiating HCM from other causes of LV hypertrophy [3, 8, 13].

Additionally, contrast-enhanced cardiac MR with late-gadolinium enhancement (LGE) has the ability to identify areas of myocardial fibrosis/scarring that may be at increased risk for SCD, and it has implications for management strategies, such as primary prevention implantable cardioverter defibrillator (ICD) therapy [3, 8, 13–15].

The stages of HCM, which are based on the clinical evidence of disease progression, were previously defined by Olivotto et al. [16]. These clinical stages include subclinical HCM, the classic HCM phenothype, adverse remodelling and overt dysfunction, or end-stage HCM.

The aim of this article is to illustrate and review the contributions of cardiac MRI for the assessment of morphological and functional consequences of different stages of HCM (Table 1).

**Table 1:** Cardiac MR characteristics of different stages of HCM

	Cardiac MR imaging				
		Late-gadolinium enhancement (LGE)			
HCM stage	Morphological and functional features	Prevalence	% / LV mass	Location	SCD risk/year
Subclinical	Normal/borderline myocardial hypertrophy Myocardial crypts Mitral valve leaflets elongation Normal LA diameter Myocardial trabeculations Myocardial fibrosis Normal LV EF	Rare	Limited	Mid-wall	Exceptional
Classic (75%)	Myocardial hypertrophy LVOT obstruction (70%) Mid or moderate LA dilatation Diastolic dysfunction LV EF > 65%	< 50%	2%	Mid-wall	0,5–1%
Adverse remodelling (15–20%)	Progressive LV wall thinning Reduction/loss of LVOT Moderate/severe LA dilatation Diastolic dysfunction LV EF: 50–65%	> 50%	10–15%	Mid-wall/transmural	3–5%
End-stage (5–10%)	Dilated form Restrictive pattern LV EF < 50%	75–100%	> 25%	Mid-wall/transmural	10%

*LV* left ventricle, *EF* ejection fraction, *SCD* sudden cardiac death, *LA* left atrium, *LVOT* left ventricular outflow tract

## Subclinical HCM

Subclinical HCM refers to the detection of subjects who carry any stigmata of HCM causing gene mutation but who are without LV hypertrophy (genotype-positive, phenotype-negative subjects). In these patients, the risk for SCD is exceptional [9].

When genetic testing is negative or ambiguous (30 to 40% of patients), cardiac MR can demonstrate a degree of LV hypertrophy that is not fully appreciated by echocardiogram [8] and other abnormalities, such as myocardial crypts, elongated mitral valve leaflets and apical myocardial trabeculations [8–13], expanded extracellular space (with T1 mapping) and LGE [17, 18]. These individuals should be periodically followed for the development of an increased LV wall thickness [4, 5, 19].

### Myocardial crypts

Myocardial clefts or crypts are congenital abnormalities related to myocardial fibre or fascicle disarray and have been described in healthy volunteers, as well as those with classic HCM (< 5%) [8, 20, 21].

**Fig. 1:**
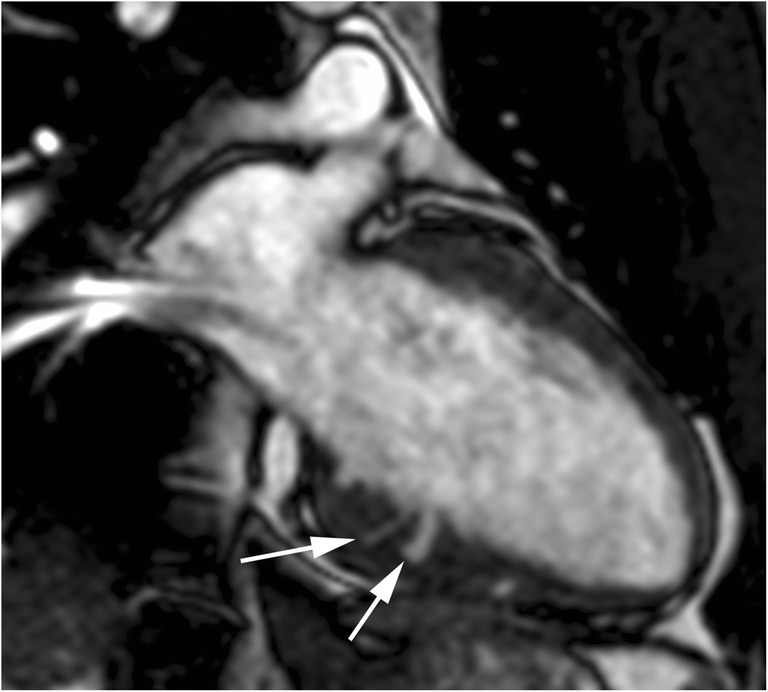
22-year-old man with myocardial crypts and a family history of HCM without myocardial hypertrophy. Two-chamber steady-state free precession (SSFP) cine MR image at end-diastole shows V-shaped myocardial crypts perpendicular to the endocardial border at inferior LV wall (arrows). The crypts close completely during systole (not shown)

These crypts are V- or U-shaped blood-filled myocardial invaginations perpendicular to the endocardial LV edge that penetrate more than 50% of the compact myocardium at end-diastole and collapse at end-systole (Fig. 1) [10, 21, 22]. Usually, myocardial crypts are multiple and located in the basal inferoseptal and inferior LV wall at the sites of right ventricle (RV) insertion [9, 22].

These myocardial crypts should be differentiated from trabeculations characteristic of LV noncompaction, congenital LV diverticula and aneurysms.

Unlike crypts, trabeculations of LV noncompaction are parallel to the endocardial border and do not penetrate the normal compacted myocardium [22].

Congenital LV diverticula are characterised by an outpouching that contains endocardium, myocardium, and pericardium, and they are a narrow connection to the cavity and collapse at end-systole. The primary diagnostic feature to differentiate diverticula from crypts is that the congenital diverticula extend outside the epicardial border while crypts remain confined inside the myocardial margin [23].

Congenital LV aneurysm represents an outpouching containing endocardium, epicardium and thinned myocardium that shows akinetic or dyskinetic motion. A thinned myocardium shows LGE without pericardial enhancement [23, 24].

### Mitral valve leaflets elongation

Several echocardiographic studies have reported that the mitral valve is elongated in HCM, particularly in the obstructive form of the disease [25, 26].

**Fig. 2:**
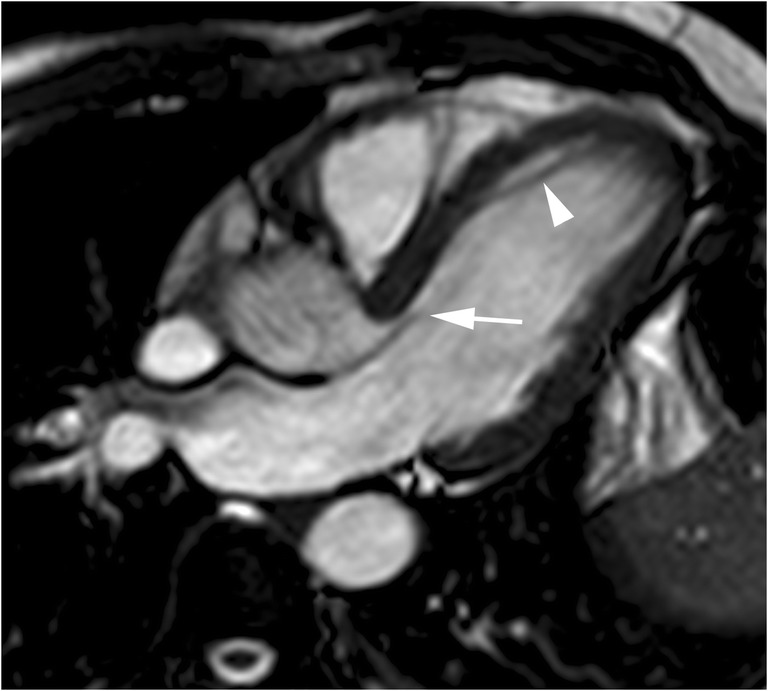
20-year-old man, genotype positive, phenotype negative. End-diastolic horizontal long-axis SSFP cine MR image demonstrates an elongated anterior mitral valve leaflet (arrow) and normal length of posterior valve leaflet. A linear structure (arrowhead) that connects ventricular septum and left ventricular apex, corresponding to aberrant muscular band also can be seen

Cardiac MR studies have shown elongated anterior and posterior mitral valve leaflets in patients with non-genotyped HCM compared to control subjects [11] and elongated anterior mitral valve leaflets in subjects with HCM-causing mutations without LV hypertrophy (Fig. 2) [11, 12, 27]. These studies suggest that anterior mitral valve leaflet elongation in young preclinical HCM patients without other phenotypic expressions of HCM represents an independent and primary component of HCM disease expression independent of age, LV thickness or the presence of LV outflow tract obstruction.

Other recent studies suggest that mitral valve elongation does not constitute the primary phenotypic expression of HCM and that the elongation of mitral valve leaflets in HCM seems to be associated with body size and left ventricular remodelling [28].

### Myocardial trabeculations

Left ventricular non-compaction is a genetically heterogeneous disorder and shares genetic mutations with HCM. Both entities can coexist and have been described as being associated with mutations in different sarcomeric genes [29].

**Fig. 3:**
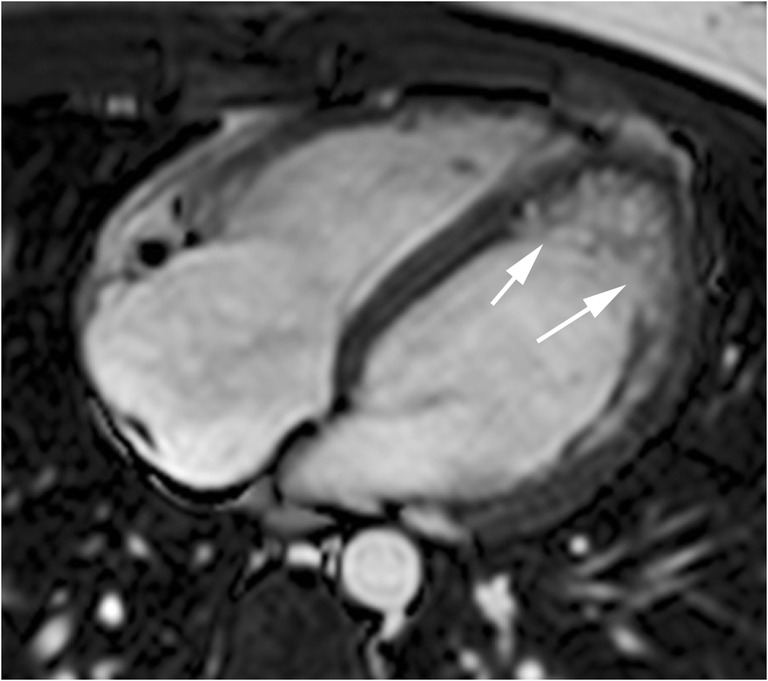
22-year-old man with a family history of HCM, genotype positive, phenotype negative. End-diastolic SSFP cine MR images showing prominent trabeculations in the LV apex (arrows) and normal miocardial thickness. These MRI features are indistinguishable from LV non-compaction

Abnormal LV apical myocardial trabeculations also can be detected in HCM sarcomere gene mutation carriers without evident hypertrophy (Fig. 3). Both diseases can have the same mutation of myocardial sarcomere, and they are indistinguishable [12]. The mechanism for these abnormal trabeculations remains unclear, similar to myocardial crypts, they could represent the persistence of the embryological form into adulthood [10, 12].

### Myocardial fibrosis

The development of fibrosis is an important step in the LV remodelling process of HCM. Recent studies have demonstrated that HCM genotype-positive and phenotype-negative patients have increased levels of biomarkers of collagen deposition. This finding indicates that increased levels of profibrotic markers are also found in patients without ventricular hypertrophy, suggesting that hypertrophy is preceded by the fibrosis process. In genotype-positive, phenotype-negative HCM subjects, several cardiac MR studies found that the myocardial mid-wall LGE consistent with fibrosis was attributed to episodes of myocardial ischaemia due to coronary microcirculation remodelling [18]. Other studies have shown that both native T1 values and extracellular volume (ECV) fraction obtained after gadolinium administration can detect diffuse interstitial fibrosis and are elevated in regions without LGE in subjects with a mutation known to cause HCM without sufficient phenotypic expression to diagnose the disease based on current imaging criteria [17].

Native T1 mapping and the ECV fraction could be used at the initial phase of HCM for the early detection of interstitial diffuse myocardial fibrosis in these patients and to monitor disease progression, as well as to evaluate novel disease-modifying therapy, targeting interstitial fibrosis [8, 17].

### Differential diagnosis

The diagnosis of mild HCM in young competitive athletes may be challenging when the LV wall thickness is between 13 and 15 mm (or 12 and 13 mm in women), which identifies the “grey-zone” of overlap between the physiological adaptations to training and mild phenotypic expression of the disease. In such instances, the diagnosis can often be resolved by applying noninvasive markers. HCM is favoured with the identification of a pathogenic sarcomere mutation or a family history of HCM, an abnormal LV filling/relaxation, small/normal size LV, myocardial crypts or LGE on contrast cardiac MR and response to detraining [30, 31].

Recent studies have shown that native T1 values and myocardial ECV by T1 mapping can be used in the differential diagnosis between HCM and an athlete’s heart. While the ECV fraction increases with increasing LV hypertrophy in HCM (due to extracellular matrix expansion and myocardial disarray), the ECV fraction reduces in athletes with an increasing wall thickness (due to an increase in the healthy myocardium by cellular hypertrophy) [32].

## Classic HCM

The classic and most common HCM phenotype (75%) consists of a hypertrophied, nondilated and hyperdynamic LV (ejection fraction > 65%), which arises during adolescence and is usually complete by young adulthood, though the onset of its phenotype may occur at virtually any age, including in utero and in those older than 60 years [33].

### Distribution of hypertrophy

**Fig. 4:**
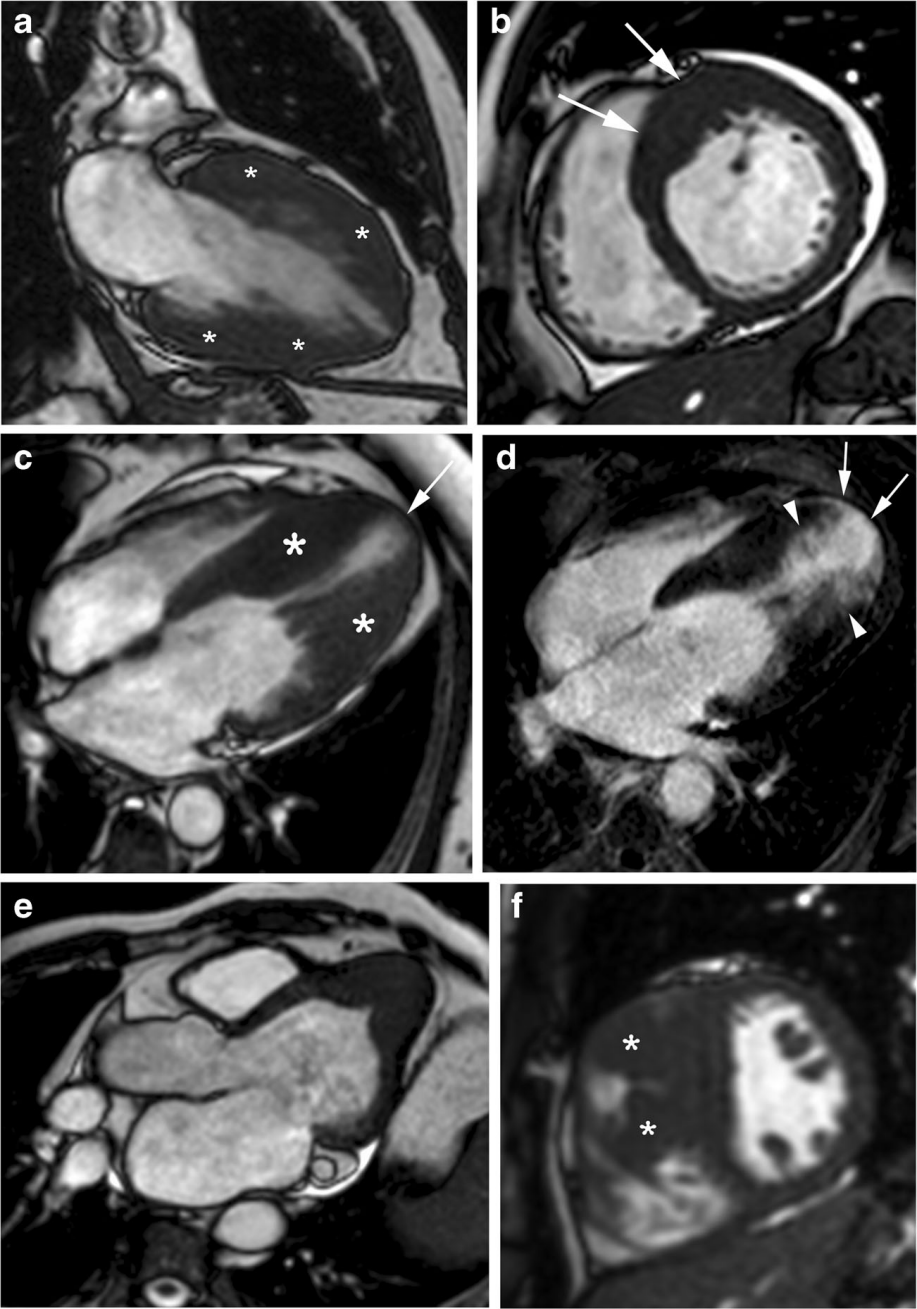
Distribution of myocardial hypertrophy in classic HCM phenotype. **a**, 56-year-old man with symmetric myocardial hypertrophy. End-diastolic two-chamber SSFP cine MR image shows diffuse and symmetric LV wall hypertrophy (asterisks); **b**, 43-year-old man who presented with palpitations. Short-axis SSFP cine MR image at end-diastole shows asymmetric septal wall hypertrophy (arrows); **c**, 57-year-old man with severe dyspnea. End-diastolic four-chamber SSFP cine MR image demonstrates a “dumbbell” configuration of the LV cavity with midventricular myocardial thickening (asterisks), marked muscular midcavity systolic constriction and a thin-walled apical aneurysm (arrow). **d**, Four-chamber LGE MR image clearly depicts the enhanced thinned apical LV aneurysm (arrows) extending from the aneurysm rim into the septum and free wall (arrowheads). Global systolic function also decreased because the ejection fraction was 35%; **e**, 53-year-old woman with apical HCM. End-diastolic horizontal long-axis SSFP MR image demosntrates the spade-like appearance of LV chamber secondary to apical LV hypertrophy; **f**, 22-year-old man with right ventricular hypertrophy. Short-axis SSFP cine MR image at end-diastole shows a prominent right ventricular structure that inserts directly on the anterior ventricular septum corresponding to large hypertophied crista supraventicularis muscle (arrowheads)

The distribution of myocardial hypertrophy is extremely variable. Cardiac MRI is particularly useful for characterising the location and extent of LV hypertrophy (Fig. 4), offering a superior visualisation and a higher diagnostic accuracy compared with 2D echocardiography, particularly if the involved segments are the basal anterolateral free wall or the apex [34, 35].

#### Left ventricular hypertrophy

The most common location of LV hypertrophy is the confluence of the basal anterior septum with the contiguous anterior free wall (70%) (Fig. 4b) [3, 8, 35]. The next most common segment with increased wall thickening is the posterior septum at the mid-LV level [27, 35].

The extent of hypertrophy is focal (involving fewer than three segments), intermediate (3–7 segments) or most common diffuse (more than seven segments) (Fig. 4a). Patients with LVOT obstruction shows hypertrophy of more segments [35]. Massive hypertrophy is considered when the LV wall thickness is > 3 cm and it has important prognostic considerations. However, in approximately 20% of HCM patients, the LV mass index values are normal on cardiac MRI [8, 19].

Mid-ventricular HCM shows predominant hypertrophy at the mid-ventricular level. Patients with this type of HCM constitute an unusual and important group of patients because severe mid-ventricular HCM can cause ventricular arrhythmia, myocardial necrosis, and systemic embolism secondary to dynamic obstruction associated with apical aneurysm (Fig. 4c) [3, 36]. Contrast-enhanced cardiac MR has demonstrated that the aneurysm rim in these patients is composed predominantly of fibrosis that extends from the aneurysm rim into the septum and free wall (Fig. 4d) and serves as nidus for ventricular tachycardia [36].

Apical HCM (5–25%) shows a predominant apical distribution of hypertrophy with a characteristic spade-like configuration of the LV cavity and is usually associated with giant inverted anterolateral T-waves on the electrocardiogram (Fig. 4e) [34]. The apical extension of LV hypertrophy is found in 10% of classic septal HCM patients, suggesting that both phenotypic expressions may coexist [8].

#### Right ventricular hypertrophy

Hypertrophy of the RV wall and RV muscle structures, such as that of the crista supraventricularis are common (Fig. 4f). Most HCM patients (53%) have diffuse RV hypertrophy involving all three segments of the RV, but a conspicuous proportion (47%) demonstrate RV hypertrophy confined to only the segments contiguous with the ventricular septum [37].

### Diastolic dysfunction

In HCM, reduced ventricular volumes are frequent and the hyperkinetic appearance of systolic contraction translates into a normal or supernormal ejection fraction (EF) (> 65%) until the end-stage of the disease [8]. EF is, therefore, considered inadequate to evaluate the indication for medical treatment and cardiac transplantation [2].

Diastolic dysfunction is one of the early hallmark features of HCM, and it is related to myocardial disarray and fibrosis, even in the absence of LV hypertrophy [8, 38].

Doppler parameters are accurate in evaluating diastolic function in HCM. Accurate measurements of transmitral and transpulmonary vein flow parameters using phase-contrast cardiac MRI are lower than those echocardiography, which is probably due to the lower temporal resolution of the breath-hold phase-contrast sequence. A new semi-automated, three-dimensional model-based analysis of the LV fillings curve showed a similar agreement with Doppler parameters [39].

In addition, myocardial tagging [39, 40] enables the quantification of cardiac strain evolution during late diastole, which can be used to assess diastolic myocardial dysfunction [40]. However, despite recent advances of myocardial tagging methods, the fading of taglines due to T1 relaxation is a common inherent limitation of all existing methods, which also requires prolonged imaging acquisition and post-processing times [39].

### Mitral valve apparatus

Abnormalities in the morphology and function of the mitral valve apparatus are now considered a phenotypic expression of HCM that is independent of the hypertrophy distribution [11].

**Fig. 5:**
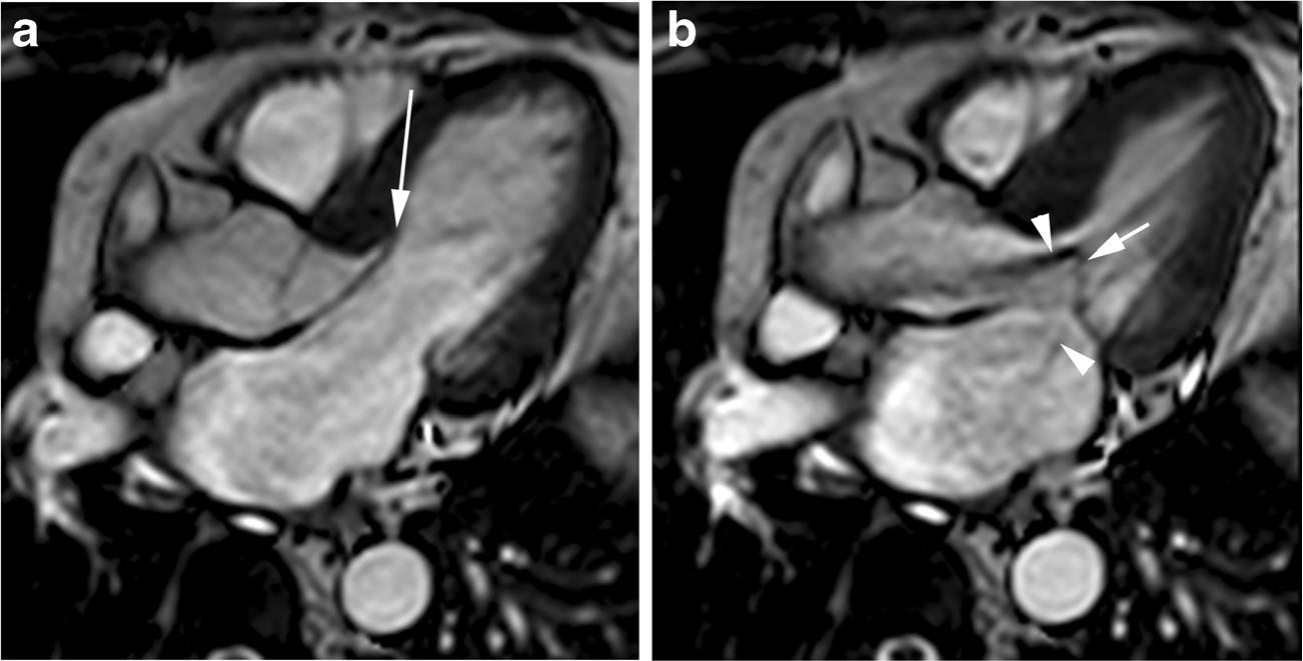
Abnormalities of mitral apparatus in classic HCM phenotype. 44-year-old woman with obstructive HCM, systolic anterior motion of the anterior leaflet of the mitral and mitral regurgitation. Horizontal long-axis SSFP cine MR images at (**a**) end-diastole and (**b**) mid-systole show an elongated anterior mitral leaflet (large arrow) with anterior displacement (small arrow) at mid-systole and turbulent-velocity jets (arrowheads) within the LV outflow tract and into the left atrium due to LV outflow tract obstruction and mitral regurgitation, respectively

Mitral valve leaflets are increased in length in many HCM patients, and substantially elongated mitral valve leaflets are an important determinant of LVOT obstruction in some patients (Fig. 5) [8, 41].

**Fig. 6:**
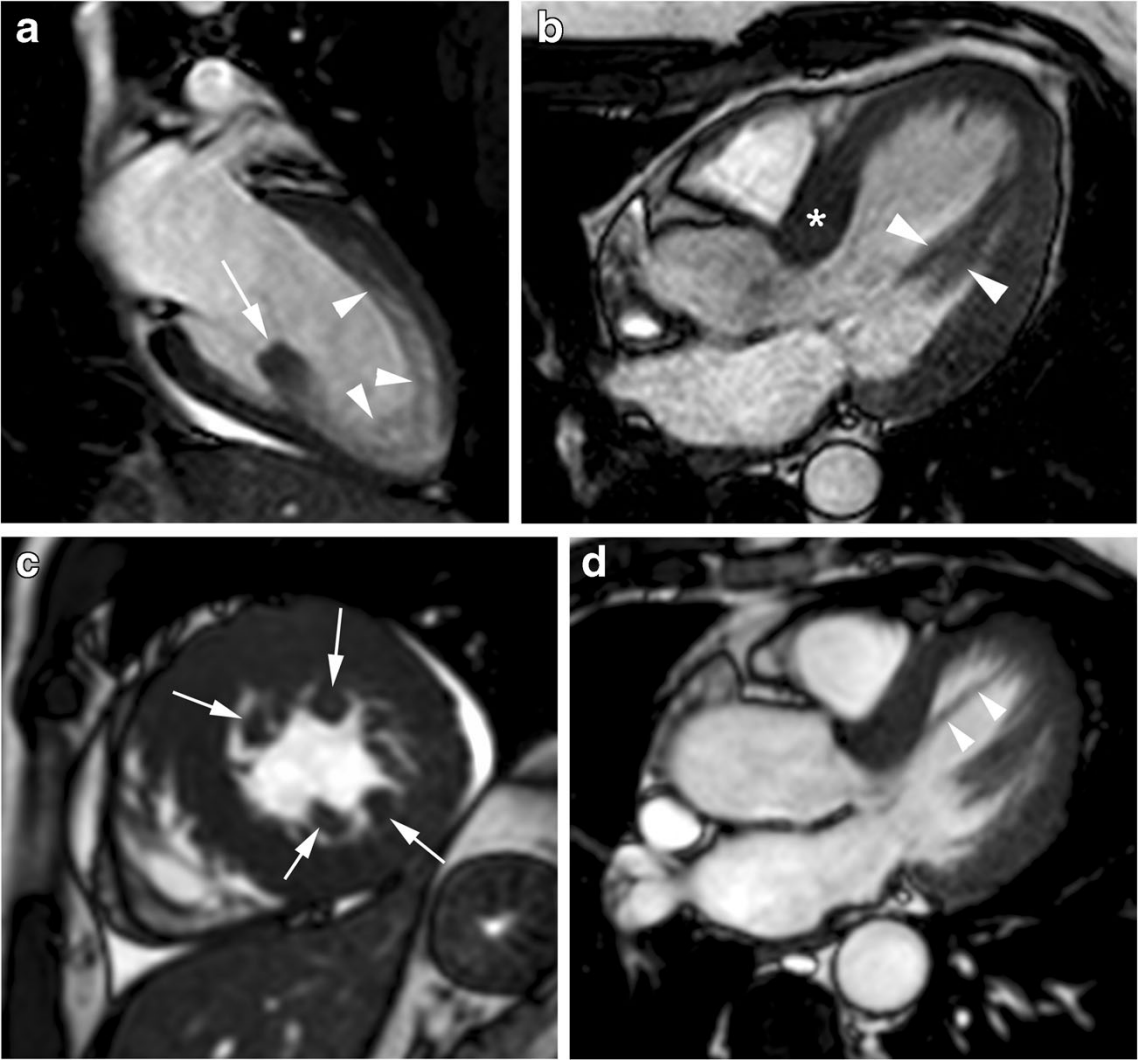
Abnormalities of papillary muscles and mitral apparatus in classic HCM phenotype. **a**, 21-year-old woman with atypical chest and electrocardiographic abnormalities suggestive of HCM). End-diastolic two-chamber SSFP cine MR image shows isolated posterior papillary muscle hypertrophy (arrow) and myocardial trabeculations along the anterior LV wall and apex (arrowheads); **b**, 45-year-old woman with asymmetric septal HCM and anomaly of papillary muscle. Horizontal long-axis SSFP cine MR image at end-diastole shows bifid anterolateral papillary heads (arrowheads) and basal septal wall thickening (asterisk); **c**, 45-year-old man with chest pain. Short-axis SSFP cine MR image at end-diastole demonstrates multiple accessory papillary muscles, four in number (arrows), diffuse LV hypertrophy and pericardial effusion; **d**, 17-year-old man with myocardial hypertrophy limited to the basal ventricular septum. Horizontal long-axis SSFP cine MR image shows accessory left ventricular apical muscle bundle (arrowheads) traversing the LV cavity from the basal septal wall to the distal portion of the chamber

Other abnormalities include hypertrophy of the papillary muscle heads, an increased number of papillary muscles, anterior and apical displacement of the papillary muscles, direct insertion of the anterolateral papillary muscle to the ventricular aspect of anterior mitral valve leaflet and LV accessory muscle bundles extending from the apex to the mid-ventricular or basal levels of the LV anterior or septal walls (Fig. 6) [42].

### LV outflow tract obstruction

LVOT obstruction is present in 70% of classic HCM phenotype patients and is associated with an increased risk for heart failure-related death [2, 8]. The mechanisms responsible for LVOT obstruction are related to complex anatomical relationships between the basal septum, LVOT, mitral valve, and papillary muscles [8, 41].

An elongated anterior mitral leaflet (Fig. 5a) is considered an important determination of LVOT obstruction, particularly in patients in whom the mitral leaflet length exceeds the transverse dimension of the outflow tract at end-systole 2-fold [3, 8, 11]. Systolic anterior motion (SAM) of the mitral valve refers to the paradoxical movement of the anterior leaflet and/or chordae toward the interventricular septum during systole. The precise mechanism of SAM in HCM remains unclear. Many investigators hypothesise that anterior mitral leaflet elongation and thickening and hypertrophy with anterior displacement of the papillary muscles contribute to the development of SAM. The resultant flow forces during systolic anterior motion pulling the leaflets into the LVOT in mid- and late-systole also result in reduced leaflet coaptation and a posteriorly directed mitral regurgitant jet (Fig. 5b) [25, 26].

Echocardiography remains the technique of choice to evaluate LVOT obstruction. However, cardiac MR-derived planimetry at the rest of the LVOT area during systole can identify obstructive HCM. A cutoff value of 2.7 cm^2^ has been reported as having an accuracy of 100% to differentiate obstructive from non-obstructive HCM [43]. Moreover, cine MRI in a long-axis cine view can provide a more accurate evaluation of the mechanism of outflow tract obstruction, demonstrating turbulent flow generated by systolic movement of the anterior mitral leaflet, chordae, and papillary muscle toward the interventricular septum [41] (Fig. 6).

### Left atrial size

The left atrium (LA) is often enlarged in HCM patients, and its size provides important prognostic information. An enlarged LA is related to an increased morbidity and mortality in cardiovascular patients, and it is considered a marker for risk for SCD and potentially lethal arrhythmic events among HCM patients [44–46].

Volumetric MR quantification is the best approach to assess the LA size, but it may be too time-consuming. An area ≧ 15 cm^2^/m^2^ and a transverse diameter ≧ 2.8 cm/m^2^ in the four-chamber view are valuable alternatives to identify LA enlargement [47].

The cause of LA enlargement is multifactorial, but the most common mechanisms are SAM-related mitral regurgitation and elevated LV filling pressures [48]. Mild or moderate LA dilatation is common and is related to LV diastolic dysfunction [44]. Severe LA dilattion is common when mitral regurgitation is associated with LVOT obstruction [8, 41].

Atrial fibrillation is the most common arrhythmia in HCM (20%) and LA remodelling is the strongest predictor of atrial fibrillation independent of age and functional class [48].

### Microvascular ischaemia

The hallmarks of HCM include LV hypertrophy, fibrosis and microvascular ischaemia, and whether there is a causative link between these features has not been determined to date [49].

Microvascular ischaemia in HCM, which is attributed to increased oxygen requirements due to hypertrophy, impaired ventricular relaxation, microvascular dysfunction of intramyocardial arterioles, and LVOT obstruction, is associated with chest pain, clinical deterioration, diastolic dysfunction, and an adverse prognosis [50, 51]. It is most pronounced in the hypertrophied segments and in gene-positive HCM patients [52].

Although microvascular dysfunction may precede clinical deterioration for several years and is present in all stages of HCM, it is not associated with evidence of permanent ischaemic damage and replacement fibrosis [16, 19].

The presence and severity of myocardial ischaemia and reduced myocardial blood flow can be diagnosed using non-invasive imaging modalities, including positron emission tomography (PET) [51], single photon emission computed tomography (SPECT) [53] and first-pass perfusion cardiac MR sequences [8, 54]. Myocardial perfusion defects are usually found at subendocardium (most common) or at mid-wall, and it correlates with the areas of maximal wall thickness [54].

### Myocardial fibrosis

Myocardial fibrosis in HCM is histopathologically diffuse in both the replacement and interstitial forms, and it is assumed to be a substrate for tachyarrhythmias and SCD. LGE essentially shows the focal distribution of fibrosis on cardiac MR, is present in less than half of those with the classic HCM phenotype and occupies a small percentage of the LV mass (median value: 2%) [16, 55], suggesting that collagen deposition at this stage reflects an exaggerated activation of the matrix rather than a reparative process [56].

**Fig. 7:**
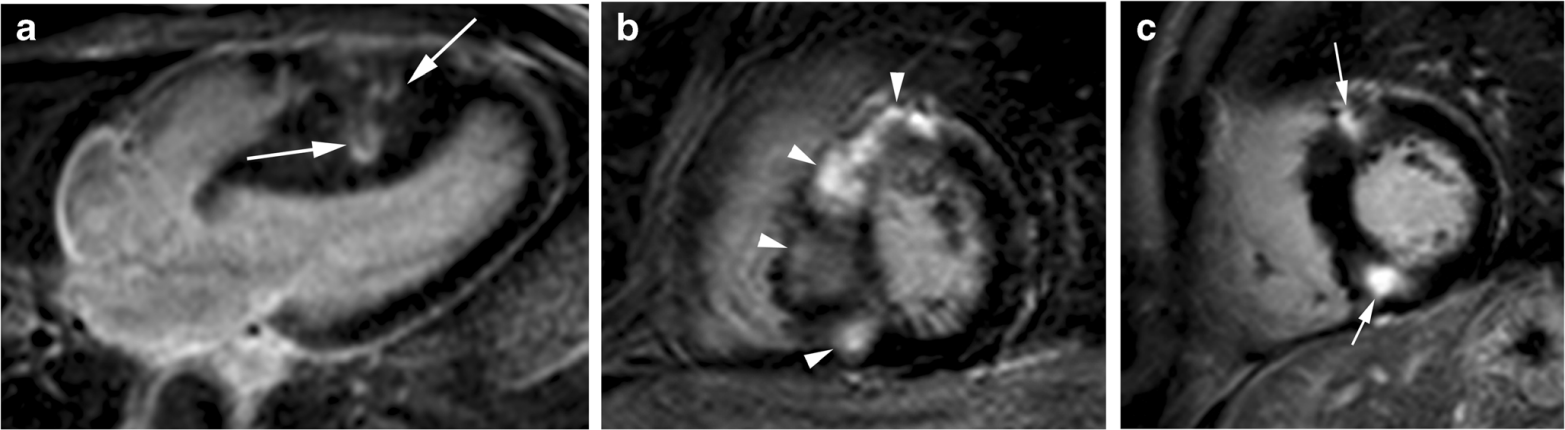
Most common distribution of LGE in classic HCM phenotype. **a**, 16-year-old man with septal HCM and recurrent ventricular tachycardia. Four-chamber LGE image shows isolated patchy mid-wall septal enhancement (arrows); **b**, 65-year-old man with palpitations and syncope and diffuse HCM. Short-axis LGE image demonstrates diffuse patchy mid-wall septal enhancement in segments with maximum LV wall thickness (arrowheads); **c**, 48-year-old man with left bundle branch block at electrocardiography and asymmetrical septal. Short-axis LGE image shows patchy delayed enhancement at insertion points (arrows) of the RV wall into the anterior and posterior ventricular septum

Myocardial LGE can present in a wide variety of patterns, although it never corresponds to a coronary vascular distribution (Figs. 4d and 7) [15, 57, 58]. It is usually localised in segments with the maximum LV wall thickness. Isolated or multiple patchy LGE at mid-wall (most common) (Fig. 7a, b) and along the RV insertion points on the septum (Fig. 7c) are the usual patterns of the classic HCM phenotype. LGE is unusual in non-hypertrophied segments [8, 59].

**Fig. 8:**
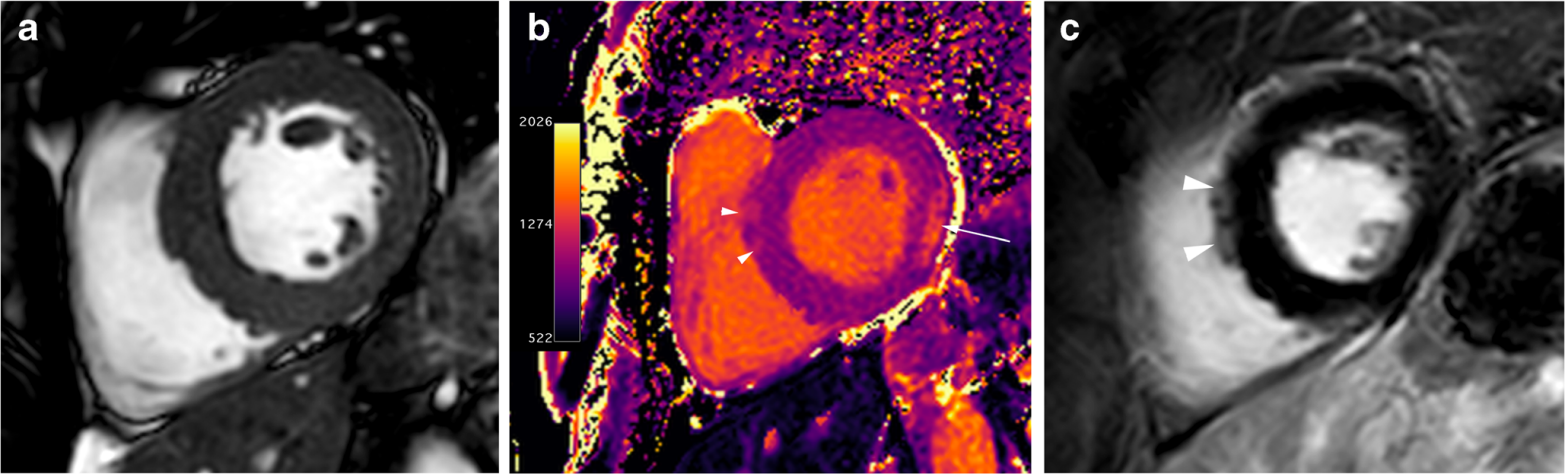
T1 mapping in a 60-year-old man with septal HCM. **a**, End-diastolic short-axis SSFP cine MR image showing septal myocardial hypertrophy (20 mm). **b**, Short-axis non-enhanced MOLLI (modified look locker inversion recovery) (1,5 T) T1 map demonstrates patchy areas of prolonged T1 values in the anteroseptal segment (arrowheads; 1203 ms) and in the lateral wall (arrow; 1343 ms). At 1.5 T, normal myocardium has a T1 relaxation time of 940–1000 ms. **c**, Short-axis LGE MR image through the same plane shows small patchy myocardial enhancement at anteroseptal segment (arrowheads)

**Fig. 9:**
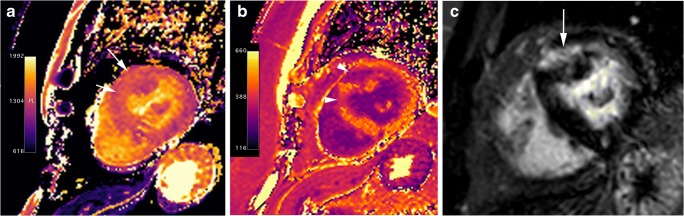
T1 mapping in a 67-year-old woman with basal anterior and anteroseptal HCM. **a**, Short-axis nonenhanced MOLLI (1.5 T) T1 map shows prolonged T1 values in the area of myocardial hypertrophy (arrows). **b**, T1 map obtained at LGE MR imaging shows abnormal shortening of the T1 values in the hypertrophied segments (arrowheads). The extracellular volume (ECV) for the hypertrophied segments was abnormally elevated (44,6 vs 29% for the lateral wall) (haematocrit, 0.42). Normal ECV values of 25.3 ± 3.5% (1.5 T). **c**, LGE MR image through the same plane shows midwall enhancement in the hypertrophied segments (arrow)

Histologically, fibrosis is often global, or diffuse, and often undetectable by standard LGE pulse sequences. T1 mapping is a novel and promising cardiac MR technique that provides an assessment of the total extent of the expanded extracellular space rather than the detection of regional areas of myocardial fibrosis identified by traditional LGE imaging. Patients with HCM have substantially higher-than-normal native T1 values and extracellular volume fractions, even in areas without LGE [60, 61] (Fig. 8), and reduced post-contrast myocardial T1 values consistent with the presence of diffuse interstitial fibrosis (Fig. 9). The imperfect concordance between native T1 mapping and LGE imaging for detecting focal replacement fibrosis may be explained by the distinct significance of both imaging methods because LGE (as well as postcontrast T1 mapping) reflects only the extracellular space, whereas native T1 mapping reflects a composite of both the intra- and extracellular compartments [62].

### Differential diagnosis

Myocardial hypertrophy can occur as a result of a variety of cardiac and systemic diseases, such as hypertensive heart disease, amyloidosis and Anderson-Fabry disease [8, 63–65].

Systemic hypertension can lead to concentric LV hypertrophy, and discriminating between HCM and hypertensive heart disease is a frequent dilemma in clinical practice. Unlike HCM, the LV wall thickness in hypertensive heart disease rarely exceeds 16 mm, and usually, there is no LGE [63].

**Fig. 10:**
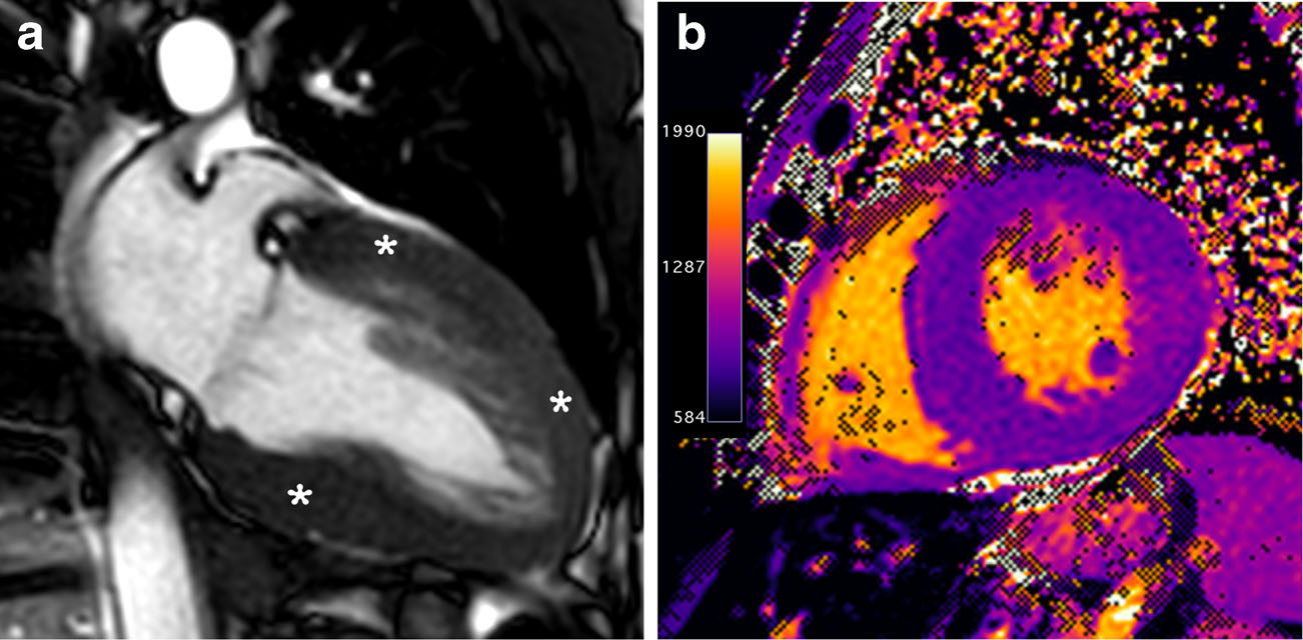
45-year-old man with gene-positive Anderson-Fabry disease. **a**, End-diastolic fouchamber SSFP cine MR image shows diffuse and symmetric LV wall hypertrophy (asterisks). **b**, Non-enhanced short-axis MOLLI native T1 map at 1.5 T shows significant shortening of T1 values (840 ms) in the hypertrophy segments. Post-gadolinium images were not obtained by renal impairment

The differentiation of Anderson-Fabry disease from HCM remains a clinical challenge, given that there is overlap in their imaging findings. Recent studies have shown that native T1 values are significantly lower in patients with Anderson-Fabry disease compared with HCM patients (Fig. 10). This is probably the most sensitive and specific cardiac MR feature to differentiate between both diseases [64].

Hypertrophy in amyloidosis is usually moderate and relatively concentric, involving both ventricles, atria, inter-atrial septum and valves. Amyloidosis displays a specific pattern of global or segmental subendocardial LGE and frequently atrial enhancement as well [8]. In addition, the typical markedly elevated native T1 values contribute to differentiating amyloidosis from HCM [65].

### Risk stratification

Although SCD is infrequent in classic HCM (0.5–1%/year), the risk stratification for SCD should be performed in all patients with HCM due to the efficacy of the ICD in preventing SCD in this disease [1, 2, 44].

The major risk factors for SCD in HCM include a family history of SCD, age (< 40 years), unexplained syncope, an abnormal blood pressure response to exercise, nonsustained ventricular tachycardia, a maximal LV wall thickness > 30 mm, a LVOT gradient ≥ 30 mmHg at rest and the LA diameter [1, 2, 44]. Although current risk factor models, such as the American algorithm [1] and European Society of Cardiology HCM Risk-SCD calculator [46] are highly effective in identifying many patients with HCM who will benefit from ICD, some patients without conventional risk markers nevertheless remain at risk for SCD [66].

Currently, neither American guidelines nor the HCM Risk-SCD calculator incorporate LGE [1, 44]. However, multiple studies have demonstrated that LGE is correlated with areas of myocardial fibrosis as the arrhythmogenic substrate for ventricular tachyarrhythmias [27, 46, 67] and might be considered a potential risk modifier, particularly when it is seen in extensive areas of the myocardium (LGE ≥ 15% of the LV myocardium) [13, 14, 66, 68]. It has been postulated that T1 mapping may prove to be superior to LGE for risk stratification in HCM. However, to date, there has been no link between T1 mapping and cardiovascular outcomes within HCM [69].

Another imaging feature with a potential impact on the current risk stratification models could be the myocardial high T2 signal intensity areas. These areas have been related to markers of adverse disease progression, such as LGE, elevated troponin and non-sustained ventricular tachycardia [70] and they have been recently implicated as a potential tool for the prediction of SCD [71].

## Adverse remodelling

**Fig. 11:**
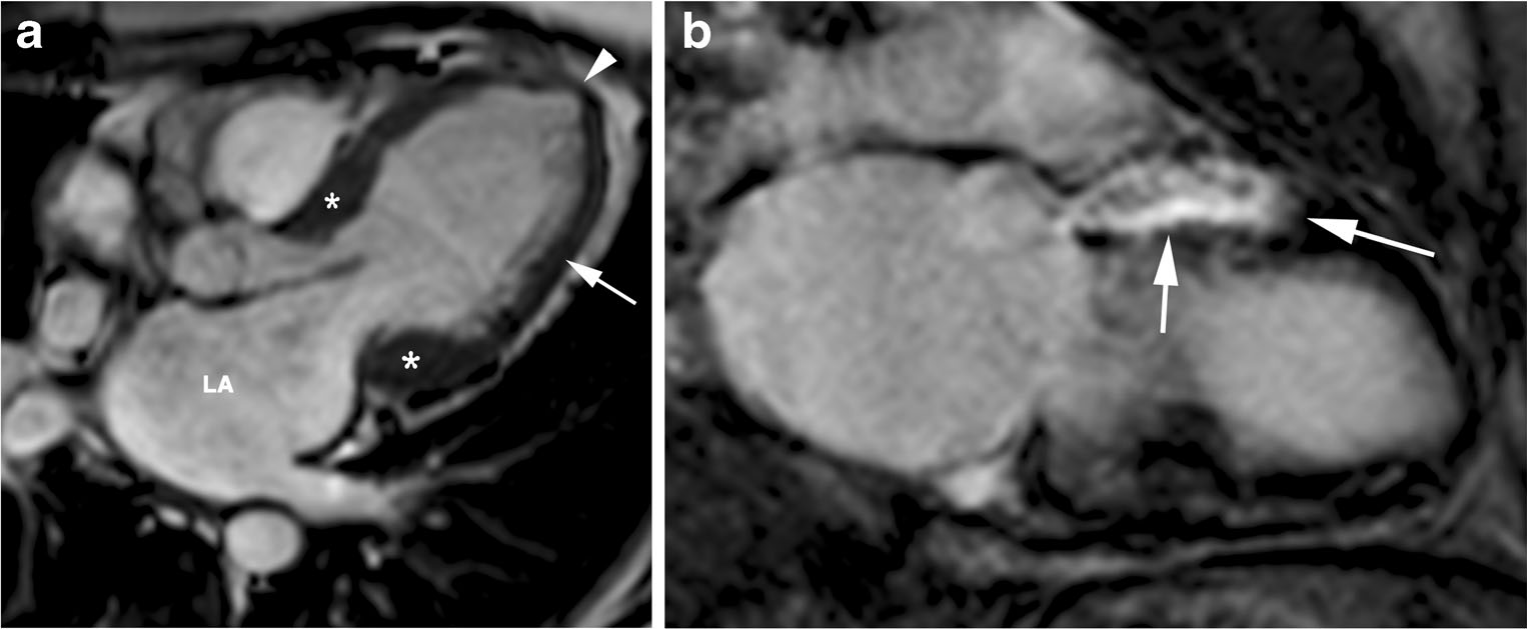
Adverse remodelling. 77-year-old man with long-standing asymmetric septal and lateral HCM who presented with increasing dyspnea on exertion and palpitations. **a**, End-diastolic horizontal long-axis SSFP cine MR image shows dilated and dysfunctional LV (ejection fraction, 45%), moderately thickened basal septum and lateral wall (15 mm) (asterisks), substantial thinning of the lateral LV wall (arrow), apical aneurysm (arrowhead) and dilated left atrium (LA). **b**, Short-axis inversion-recovery image shows transmural delayed enhancement in the anterior wall (arrows)

Adverse remodelling occurs in 15–20% of HCM patients and is characterised by progressive decreases in systolic (EF: 50–65%) and diastolic LV function [16, 55], moderate/severe LA and LV dilatation, an increase in symptoms and functional limitations [72], atrial fibrillation [38], reduction or loss of LVOT, and progressive LV wall thinning, which are thought to be due to small-vessel ischaemia (Fig. 11) [73].

Myocardial LGE is common (> 50%) [8] and is usually multiple and confluent at the mid-wall or transmural [8, 16], and the extent of LGE accounts for 10–15% or more of the LV mass [8, 14].

The risk for SCD is intermediate (3–5%/year) ranging from a low risk for the classic HCM and a high risk for the end-stage HCM [8, 15, 16].

The diagnosis of adverse remodelling is important because it can alter management strategies including the consideration of prophylactic ICD, and timely evaluation for heart transplantation, before symptoms develop [72].

## End-stage HCM

A small distinctive group of HCM patients (5–10%) will develop the high-risk phenotype of end-stage disease, which is characterised by progressive LV wall thinning, LV dilatation, a decrease or disappearance of LVOT gradients and LV systolic (EF < 50%) and diastolic dysfunction [8, 16, 62, 73].

**Fig. 12:**
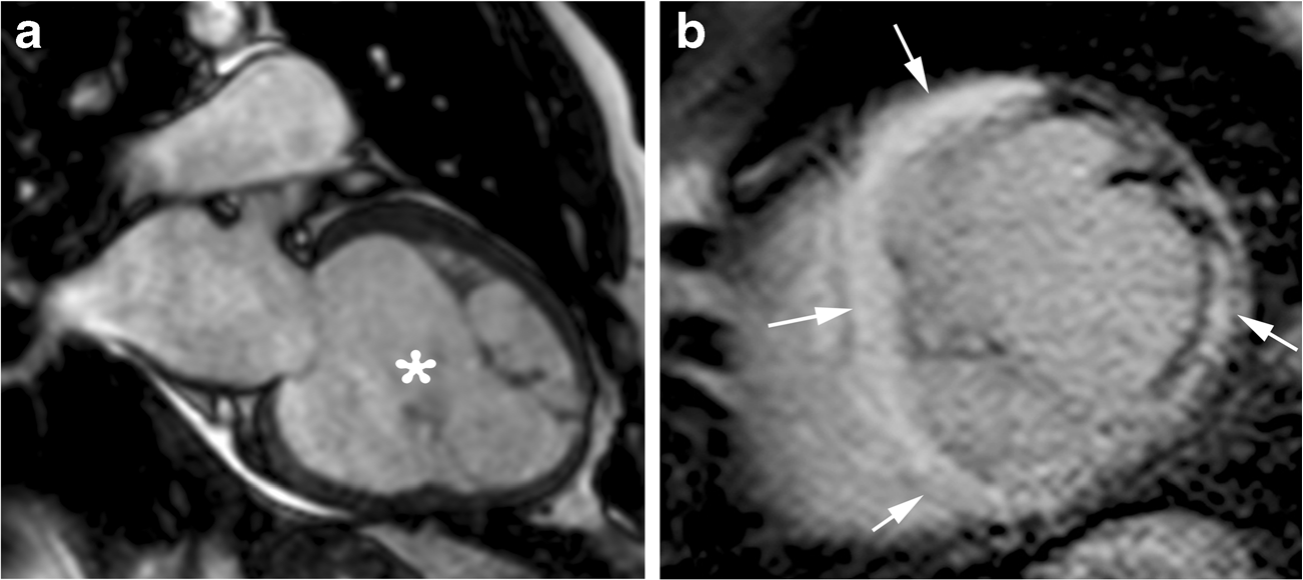
End-stage HCM with dilated form. 69-year-old man with long-standing asymmetric septal HCM who presented with dyspnea at rest and congestive heart failure. **a**, Two-chamber SSFP cine MR image shows regression of wall thickness, increase in left ventricular cavity dimensions (asterisk) and a reduced ejection-fraction of 33%. **b**, Short-axis inversion-recovery image showing transmural (arrows) delayed enhancement involving more than 25% of myocardial mass

**Fig. 13:**
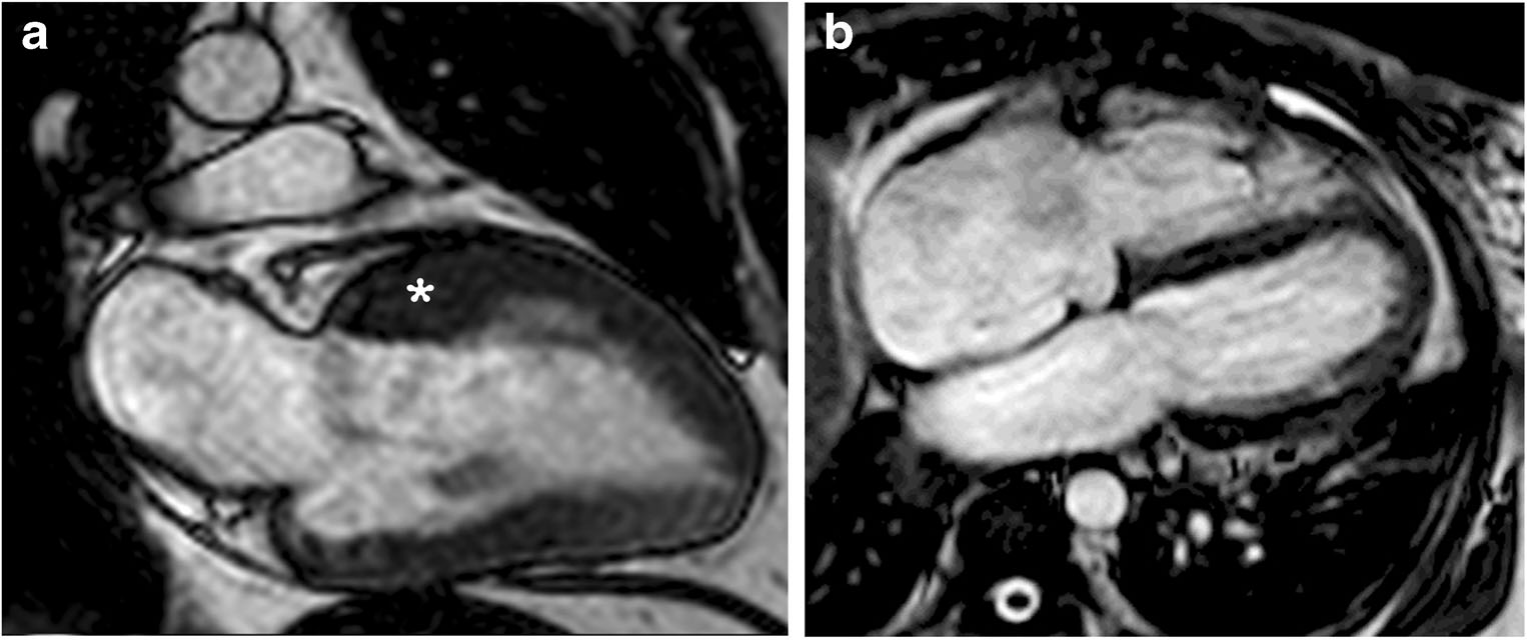
End stage HCM with restrictive pattern. 77-year-old man who presented with several months of intermittent exertional dyspnea and asymmetric HCM. He presented to the emergency room 7 years later with markedly worsening dyspnea and decompensated heart failure. **a** Two-chamber SSFP cine MR image performed at initial diagnosis shows asymmetric anterior hypertrophy (asterisk) with an ejection-fraction of 60%. **b**, Four-chamber SSFP cine MR image performed 7 years later demonstrates a small LV cavity, atrial cavities enlargement, regression of wall thickness and a reduced ejection-fraction of 37%

In certain cases, there is relative LV dilation and wall thinnings, with greater resemblance to the morphological and functional features of dilated cardiomyopathy (Fig. 12). In other cases, a restrictive pattern with a small LV and severe dilatation of both atria occurs (Fig. 13) [2, 8].

Most end-stage HCM patients show mid-wall or transmural LGE (75–100%) [8, 14]. The extent of LGE often exceeds that observed in any other cardiovascular disease (>25% of the LV mass) [27], and it is more extensive in those with ventricular remodelling and the LA is larger in those with a normal ventricular size [8, 27, 72].

This end-stage form is associated with an increased risk for SCD (by 10%/year) and heart failure-related complications [8, 16].

## Conclusions

The phenotypic expression of HCM includes a wide variety of morphological and functional manifestations that may lead to severe left ventricular wall remodelling.

Cardiac MRI provides the clinician with detailed information regarding the HCM phenotypes and enables the assessment of its functional consequences elucidating the causes and site of hypothetical dynamic obstruction, the presence and extent of myocardial perfusion abnormalities and the existence of fibrosis.

## Abbreviations

ECV: Extracellular volume
EF: Ejection fraction
HCM: Hypertrophic cardiomyopathy
ICD: Implantable cardioverter defibrillator
LA: Left atrium
LGE: Late-gadolinium enhancement
LV: Left ventricle
LVOT: LV outflow tract
MRI: Magnetic resonance imaging
PET: Positron emission tomography
RV: Right ventricle
SAM: Systolic anterior motion
SCD: Sudden cardiac death
SPECT: Single photon emission computed tomography
